# Drivers for the implementation of market‐based food safety management systems: Evidence from Lebanon

**DOI:** 10.1002/fsn3.1394

**Published:** 2020-01-17

**Authors:** Gumataw Kifle Abebe, Rachel Anne Bahn, Ali Chalak, Abed Al Kareem Yehya

**Affiliations:** ^1^ Faculty of Agricultural and Food Sciences The American University of Beirut Beirut Lebanon

**Keywords:** food processing, food safety management systems, ISO 22000, Lebanon, market orientation

## Abstract

The food safety landscape continues to evolve across time, geography, and supply chains. This research seeks to analyze the determinants of market‐based food safety management systems (FSMSs) implementation in the Middle Eastern context. Primary data were collected from food safety managers representing 94 processors across Lebanon. We found food processors having implemented ISO 22000 (50%), HACCP (40%), and ISO 9001 (25.5%); however, none of the processors implemented industry‐based FSMSs. Although ISO 22000 was mostly implemented by large (85%) and medium (67%) processors, the uptake of ISO 22000 by small processors has picked up (29%). Economic incentives (market orientation) and firm‐specific factors (organizational readiness, product/process characteristics, company size, and ownership structure) are the key drivers for the increased implementation of market‐based FSMSs. Predominantly export‐oriented processors had the odds of implementing ISO 22000 5.5 times more than the domestically oriented processors. Firms with a quality assurance (QA) unit had 15 times higher chance of implementing ISO 22000 than otherwise. Finally, processors engaged in fresh produce had 4.9 times higher chance of implementing ISO 22000 than those engaged in dry goods. The study establishes that the dominance of public‐based FSMSs in the governance of food safety is a strategic choice (economic incentives) more than statutory requirements.

## Graphical abstract

This study has sought to analyze the implementation of market‐based FSMSs in Lebanon. The study has identified public‐based international FSMSs as the dominant food safety governance regime in Lebanon. The study establishes that the dominance of public‐based FSMSs in the governance of food safety is a strategic choice (economic incentives) more than statutory requirements.

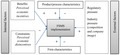

## 1 INTRODUCTION

All around the world, food safety has become a public health concern and a major development challenge (Liu, 2014; Nychas, Panagou, & Mohareb, 2016). High profile food scares related to microbiological (e.g., *Salmonella, E. coli)*, contaminant (e.g., dioxins), and animal disease (e.g., BSE) have raised a wide range of food safety legislative demands (Kendall et al., 2018). This parallels the increased complexity and globalization of food supply chains (Kotsanopoulos & Arvanitoyannis, 2017). Food companies are responding to real and perceived food safety hazards through the implementation of various food safety management systems (FSMSs) (Henson & Humphrey, 2010). Accordingly, a plethora of market‐based (nonregulatory) schemes has emerged in the last two decades. They include public‐based FSMSs, such as International Organization for Standardization (ISO) 9001, Hazard Analysis Critical Control Point (HACCP), and ISO 22000, and industry‐based FSMSs, such as GlobalGAP, British Retail Consortium (BRC), Safe Quality Food (SQF), International Food Standard (IFS), and Food Safety System Certification (FSSC 22,000). Nonetheless, the implementation of market‐based FSMSs is still far below expectations and varies across time, geography, and supply chains (Escanciano & Santos‐Vijande, 2014; Macheka, Manditsera, Ngadze, Mubaiwa, & Nyanga, 2013). FSMSs have continued to be scrutinized for their effectiveness and low uptake (Kotsanopoulos & Arvanitoyannis, 2017).

This research presents a case study of food processors in Lebanon. Strategically positioned at the center of the Eastern Mediterranean region, Lebanon provides an interesting perspective to expand our understanding of the (dis)incentives toward public‐ and industry‐based FSMSs in food supply chains originating from emerging economies. First, Lebanon serves as a commercial link between the Middle East and Europe and is an important trade partner for the European Union under the Euro‐Mediterranean partnership (Bahn & Abebe, 2017). Second, following the uncovering of many food safety scandals in 2014, food safety has gained a renewed interest in Lebanon (Abebe, Chalak, & Abiad, 2017; Massoud, Fayad, El‐Fadel, & Kamleh, 2010). Lebanon introduced its first food safety law in 2016, and it would be interesting to explore how the new law might affect the implementation of market‐based FSMSs. Prior to this law, only fragmented legislative decrees were available to address food safety issues in Lebanon. It is argued that when government oversight is strong, the adoption of market‐based FSMSs may be low because the government can provide the necessary resources to enforce food safety requirements and vice versa (Wever, Wognum, Trienekens, & Omta, 2010). The study seeks to analyze the prevalence and determinants of public‐ and industry‐based FSMSs in Lebanon, where such studies are scant (Massoud et al., 2010).

## 2 MATERIALS AND METHODS

### 2.1 Approaches for analyzing food safety

Food safety has long been studied as part of quality management (Trienekens & Zuurbier, 2008; Wever et al., 2010). However, following the uncovering of several high profile food safety scandals, food safety has become a shared responsibility among supply chain actors, consumers, and regulators (Nychas et al., 2016). Subsequently, there is now a strong need to explicitly focus on food safety and FSMSs (Hammoudi, Hoffmann, & Surry, 2009). Over the last two decades, the governance of food safety has transformed from inspection and monitoring regimes to more robust, process‐based approaches and from national food safety regulations to global, harmonized food safety standards. We argue that the harmonization of food safety systems across geographies and supply chains can enhance the implementation of international FSMSs by reducing the certification and auditing costs.

Generally, FSMSs can be classified into international (e.g., ISO 22000), industry‐based standards (e.g., BRC, IFS), and national standards (e.g., Rafeeque & Sekharan, 2018). Alternatively, FSMSs can be grouped into “public mandatory and public voluntary” or “private regulatory and private voluntary” (Henson & Humphrey, 2010, p.1631). Other studies define FSMSs based on ownership structure (private or public), scope of adoption (i.e., whether a standard covers firm‐to‐firm relations or the entire food supply chain), and scale of adoption (i.e., the extent in which an FSMS is adopted across each stage of the food supply chain) (e.g., Wever et al., 2010). This study focuses on market‐based FSMSs. A key feature of market‐based FSMSs is that their implementation is voluntary, depending on the needs of the final market (Giacomarra, Galati, Crescimanno, & Tinervia, 2016). Accordingly, the term market‐based FSMSs refers to the public voluntary systems (public market‐based FSMSs) and those standards developed by coalitions of private actors (industry‐based FSMSs) (Hatanaka, Bain, & Busch, 2005). Industry‐based FSMSs may be adopted due to the deficiency (lack) of public‐based FSMSs or as a strategic choice for product differentiation by going beyond the regulatory demands to address food safety concerns (Henson & Humphrey, 2010).

Table 1 presents the market‐based FSMSs, classified by ownership structure, scope, and scale of adoption. The public‐based international FSMSs have a greater scale of adoption than the industry‐based global FSMSs**.** This may be due to the efforts of the World Health Organization (WHO), World Trade Organization (WTO), and Food and Agricultural Organization (FAO) to harmonize food safety regulations across countries (King et al., 2017). An increasingly important public‐based FSMS is ISO 22000 (H. Chen et al., 2019). In the words of Escanciano and Santos‐Vijande (2014, p.55), ISO 22000 is “the only one that is international in character and applicable to all links in the food chain.”

**Table 1:** Overview of FSMSs implementation based on ownership structure, scope, and scale of adoption

FSMS	Ownership	Scope of adoption	Scale of adoption (# of valid certificates)
HACCP	Public (Codex Alimentarius Commission)	Across the food supply chain	Data unavailable
ISO 9001	Public (International Organization for Standardization)	All industry types	878,664 (187 countries)
ISO 22000	Public (International Organization for Standardization)	Across the food supply chain	32,120 (156 countries)
GlobalGAP	Industry; collective (Consortium of retailers run by FoodPLUS GmbH)	Primary producers	185,000 farms (125 countries)
BRC	Industry; collective (Consortium of retailers)	Food manufactures/ suppliers	17,000 (90 countries)
SQF	Industry; collective (Food Marketing Institute)	Across the food supply chain	Data unavailable
FSCC 22,000	Industry; Collective (European Food and Drink Association and the American Groceries Manufacturing Association)	Across the food supply chain	18,000 (140 countries)
IFS	Industry; collective (German, French, and Italian food business operators)	Food processing/ packaging companies	(90 countries)

**Figure 1:**
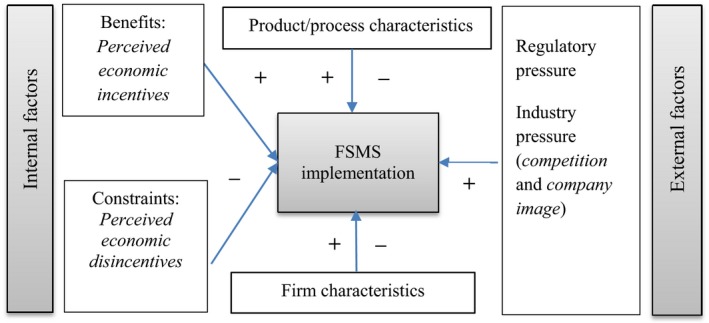
Drivers for the implementation of FSMSs: Conceptual framework

This study introduces a conceptual framework (Figure 1) about the drivers for the implementation of FSMSs. The main hypothesis is that economic (dis)incentives influence the decision (not) to implement FSMSs. This hypothesis is derived from behavioral theories that perceived benefits influence attitudes and thereby determine intentions (Ajzen, 1991). We expect that the decision (not) to implement FSMSs may be conditioned by internal factors such as financial and nonfinancial resources (Escanciano & Santos‐Vijande, 2014; Herath & Henson, 2010; Qijun & Batt, 2016) and market orientation (Chen, Flint, Perry, Perry, & Lau, 2015; Codron et al., 2014) and external factors such as regulatory demands and industry characteristics(Henson & Reardon, 2005; Wilcock, Ball, & Fajumo, 2011). Other things being equal, food processors may opt to implement market‐based FSMSs if (perceived) benefits outweigh (perceived) costs. Past studies have reported several benefits of market‐based FSMSs, including enhanced product quality and food safety (Macheka et al., 2013), efficiency (Fotopoulos, Kafetzopoulos, & Gotzamani, 2011; Massoud et al., 2010), market access (Fotopoulos et al., 2011; Macheka et al., 2013), and productivity and profitability (Fotopoulos et al., 2011).

Following Karipidis, Athanassiadis, Aggelopoulos, and Giompliakis (2009), the implementation of FSMSs can be attributed to internal and external factors. The study proposes four internal factors: Market orientation, (perceived) benefits and constraints, product/process, and firm characteristics. Perceived benefits and constraints are latent variables and are constructed based on multi‐item scales. Firm‐specific factors include organizational readiness, product/process characteristics, company size, ownership structure, and business years. Organizational readiness measures the degree of commitment by food companies (Qijun & Batt, 2016; Trienekens & Zuurbier, 2008). Two proxy variables are included to measure organizational readiness: (1) having a separate unit for food quality/safety; and (2) the management's capacity (education level of the manager) to implement FSMS. For the external dimension, regulatory measures and industry pressures, which are strongly liked to FSMSs adoption (Karaman, Cobanoglu, Tunalioglu, & Ova, 2012), are included. Failure to meet the minimum food safety requirements can lead to economic losses because of fines, product recalls, legal liability, business suspension, or loss of market competitiveness (Hammoudi et al., 2009).

### 2.2 Study context

The agro‐food sector in Lebanon generates about 32% of the industrial output, employs 25% of the industrial workforce, involves 22% of the industrial enterprises, and accounts for about 21% of total exports (IDAL, 2017). The majority of food establishments in Lebanon are small and medium enterprises (Massoud et al., 2010) and engaged in the production of baked goods (23%), confectionery (16%), dairy products (8%), grain processing (7%), fruits and vegetables (4%), oils (4%), and meat and fish (4%).

**Figure 2:**
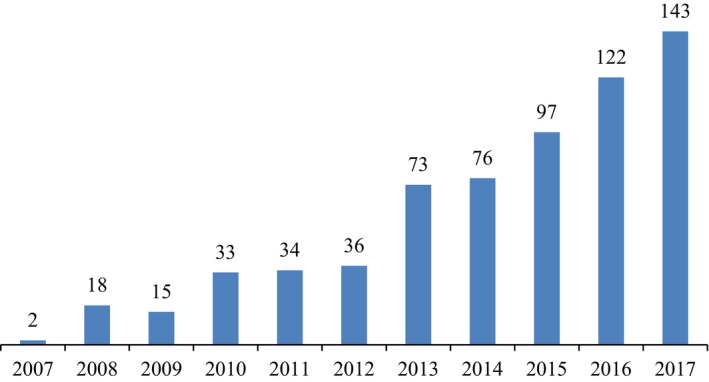
Number of ISO 22000 certificates in Lebanon

The first ISO 22000 certification to a Lebanese food establishment was awarded in 2007, and food establishments seeking for ISO 22000 certification has picked up since 2013 (Figure 2). This period matched the uncovering of several food safety scandals in Lebanon which forced the Lebanese Ministry of Health to launch a series of inspection campaigns targeting several establishments (Al‐Akhbar, 2014; Obeid, 2014). These scandals gained the attention of popular news media in Lebanon and increased public pressure and eventually led the Lebanese parliament to approve its first long‐awaited food safety law (Law No. 35) in 2016. Up until 2016, no law had covered food safety issues in Lebanon; rather food safety issues had been addressed through nine government agencies. As a result, food safety legislative decrees were fragmented and limited in scope. The main outcome of this law was the formation of the Food Safety Lebanese Commission (FSLC) whose main task is to spearhead the implementation of the food safety law at the various stages of the food supply chain.

### 2.3 Sampling procedures and data

Based on the addresses available on the Lebanese Export Directory, 342 food processors across Lebanon were invited for the survey. A structured questionnaire, approved by the Institutional Review Board (IRB) at the American University of Beirut (AUB), was used for data collection. The survey questionnaire included: company demographic information; inventory of FSMSs; market orientation; and (perceived) benefits and constraints for implementing FSMSs. A face‐to‐face interview and an online survey (via AUB‐supported Limesurvey) were conducted with the quality/food safety managers of each company. A total of 124 processors participated, but responses from 30 processors were excluded due to incomplete information. The final analysis included the responses of 94 food processors (response rate of 27.5%). A response rate of above 20% is generally considered satisfactory in studies involving business organizations (Malhotra & Grover, 1998). The surveyed companies were engaged in the processing of baked goods, meat, dairy, fruits and vegetables, confectionery, cereals, and oils. They represented all governorates of Lebanon.

## 3 RESULTS AND DISCUSSION

### 3.1 Overview of market‐based FSMSs implementation in Lebanon

Table 2 presents an overview of FSMSs implementation in Lebanon by company size. Following Escanciano and Santos‐Vijande (2014), company size was defined into small (50 or fewer employees), medium (51–250 employees), and large (more than 250 employees). Accordingly, 51% (48) of the processors were small size, and the remaining 49% were medium or large size. Most of the processors were family‐owned (68%) and had been in the business for 20 years or more (71%). Also, approximately two‐thirds of the food processors were engaged in baked goods, confectionery, cereals, and oils processing, while the remaining 37% were primarily engaged in meat, dairy, or fruits and vegetable processing.

**Table 2:** Overview of market‐based FSMSs implementation by company size (# employees, full‐time equivalent)

	*N*	Implemented (*n* = 47)	Not implemented (*n* = 47)	Chi‐square test
*PRPs*				
GMP/GHP^a^				
Company size				
Small (50 or less)	48	13 (27%)	35 (73%)	11.4 (*df* = 2, *p* < .003)
Medium (51–250)	33	19 (58%)	14 (42%)	
Large (>250)	13	9 (69%)	4 (31%)	
SSOPs^b^				
Company size				
Small (50 or less)	48	10 (21%)	38 (79%)	6.4 (*df* = 2, *p* < .04)
Medium (51–250)	33	13 (39%)	20 (61%)	
Large (>250)	13	7 (54%)	6 (46%)	
*Public‐based FSMSs*				
HACCP^c^, *				
Company size				
Small (50 or less)	48	9 (19%)	39 (81%)	12.4 (*df = *2*,p < .*002)
Medium (51–250)	33	33 (38%)	67 (34%)	
Large (>250)	13	9 (69%)	4 (31%)	
ISO 9001^c^, *				
Company size				
Small (50 or less)	48	2 (4%)	46 (96%)	22.6 (*df = *2*, p < .*000)
Medium (51–250)	33	10 (30%)	23 (70%)	
Large (>250)	13	8 (62%)	5 (38%)	
ISO 22000				
Company size				
Small (50 or less)	48	14 (29%)	34 (71%)	18.23 (*df = *2*, p < .*000)
Medium (51–250)	33	22 (67%)	11 (33%)	
Large (>250)	13	11 (85%)	2 (15%)	
Industry‐based FSMSs: GlobalGAP, BRC, IFS, SQF				
Company size				
Small (50 or less)	48	‐	48	‐
Medium (51–250)	33	‐	33	
Large (>250)	13	‐	13	

a Good Manufacturing/Hygiene Practices; Sanitation Standard Operating.

b Sanitation Standard Operating Procedures.

c Included companies that did not have third‐party certification (nine for HACCP and four for ISO 9000 series).

The results showed FSMSs (HACCP and ISO 22000) and the quality management system (ISO 9001) as the dominant public‐based systems implemented by the surveyed processors. In fact, none of the surveyed processors had implemented any of the industry‐based FSMSs. Half of the surveyed processors reported having implemented ISO 22000. As shown in Table 2, the chi‐square test was applied to compare the subsamples of the food processors and their size. In both subsamples, there are small, medium, and large size food processors. The implementation of the pre‐requisite programs (PRPs) and FSMSs greatly vary by and increase with company size. For example, the implementation of PRPs among the small processors was only 27% (GMP/GHP) and 21% (SSOPs) while that of the large processors was 69% (GMP/GHP) and 54% (SSOPs). Approximately 19% and 69% of the small and large processors, respectively, had applied HACCP. Small processors had an increased level of participation in the implementation of ISO 22000 (29%) compared to their level of participation in ISO 9001 (4%) and HACCP (19%). The vast majority of the large (85%) and medium (67%) processors reported having implemented ISO 22000.

### 3.2 Incentives and barriers for the implementation of market‐based FSMSs

Based on a review of recent studies on potential incentives and barriers for the implementation of FSMSs (Escanciano & Leticia Santos‐Vijande, 2014; Karaman et al., 2012; Macheka et al., 2013), 29 items (reported in Table 3) were identified and included in the questionnaire. The food safety/quality managers were asked to indicate their level of (dis)agreement on each of the 29 items (on a 7‐point Likert scale), ranging from 1 (“strongly disagree”) to 7 (“strongly agree”). The managers were informed to respond to the questions based on their experience (if implemented any of the FSMSs) or perception (if the company did not implement any FSMSs at all) (Table 3).

**Table 3:** Potential barriers and incentives for the implementation of ISO 22000 (mean scores)

Barriers to implement ISO 22000 (1 = strongly disagree; 7 = strongly agree)	Implemented (*n* = 47)	Did not implement (*n* = 47)	Chi‐square (*p‐*value)
Expensive and complicated task (i.e., there are economic, technological, and legislation constraints)	4.40	5.28	c, *
Lack of complete, accurate, timely, and easily accessible information about the need for FSMS	3.34	3.74	
Resource‐intensive, require much administration and paper works which place a burden on companies	4.57	5.36	c, *
Lack of trained staff for technical and management aspects of FSMS	3.77	4.49	c, *
Lack of willingness by other supply chain partners to participate in the implementation of FSMS	4.57	5.13	
Lack of clarity about the benefits to be gained from implementing FSMS vis‐à‐vis required investment costs	3.68	4.62	c, *
Not required by (non) governmental agencies	3.85	4.49	
Not familiar to customers and consumers	3.17	3.62	
Incentives to implement ISO 22000 (1 = strongly disagree; 7 = strongly agree)			
Reduces product losses	6.36	6.17	
Streamlines paperwork	5.70	5.72	
Avoids duplication between processes	6.26	6.45	
Increases operational efficiency	6.15	6.30	
Improves quality of management	6.55	6.70	
Enhances export competitiveness	6.36	6.43	
Avoids negative media attention	5.85	5.70	
Increases profit margins	5.79	5.79	
Improves our relationship with suppliers	5.98	5.81	
Attracts new customers	6.43	6.49	
Enhances market leadership	6.28	6.47	
Improves our relationship with customers	6.28	6.53	
Improves market share	6.32	6.28	
Accesses to new markets	6.38	6.51	
Provides competitive advantage	6.45	6.40	
Improves company image	6.64	6.70	
Reduces legal liability	6.02	6.32	
Meets customers	6.32	6.36	
Reduces risk of product recalls	6.57	6.62	
Provides evidence of legal compliance	6.53	6.43	
Provides customer assurance	6.34	6.45	

* *p* < .1; ^**^ *p* < .05; ^***^ *p* < .01.

As displayed in Table 3, significant differences exist regarding (perceived) barriers between the two subsamples that did and did not implement ISO 22000. The mean scores for the latter were greater than the mean scores of the former across all the eight items; meaning, companies that had implemented ISO 22000 did not consider those factors constraining as much as their counterparts did. However, both groups of companies were in agreement concerning the (perceived) benefits of implementing ISO 22000. Most importantly, the quality managers from both subsamples agreed that the implementation of ISO 22000 would lead to enhanced efficiency, competitiveness, and compliance with regulatory and customer demands.

Responses of the multi‐item scales were subjected to factor analysis, using SPSS (version 25.0). Initially, the study included all the 29 items; those items that scored less than 0.60 and had cross‐loadings of more than 0.35 were dropped (Varshneya & Das, 2017). A total of 24 items with a factor loading of 0.60 or higher were retained. First, Kaiser–Meyer–Olkin (KMO) and Bartlett's tests were performed on a dataset consisting of the 24 items to determine its validity for factor extraction. KMO value and Bartlett's tests are reported at 0.856 and 2,173.875 (*p* < .0001), respectively; the test results warranted a factor analysis. Second, the multi‐item scales were subjected to exploratory factor analysis, and an orthogonal rotation method, varimax, was used for factor rotation. The analysis resulted in a three‐factor solution with each dimension having an eigenvalue of greater than one and a factor loading of greater than 0.60. The three factors jointly explained 68.91% of the variance and are named “(Perceived) constraints,” “Efficiency gain,” and “External pressure.” These dimensions are consistent with an earlier study by Escanciano and Santos‐Vijande (2014). Next, reliability and validity tests were performed. Cronbach's alpha was used to measure the internal consistency reliability of the measurement scale, and all the three constructs exceeded the 0.70 threshold. All the multi‐item scales were based on extensive reviews of prior studies on FSMSs adoption (Escanciano & Leticia Santos‐Vijande, 2014; Karaman et al., 2012; Macheka et al., 2013). Thus, the scale items have content validity. Regarding, the construct validity, factor analysis was run, and each item has a high factor loading of above 0.60 (Table 4). Also, the composite reliability values of the three constructs were calculated—(perceived) constraints (0.889), efficiency gain (0.921), and external pressure (0.965)—and all the values are greater than the recommended value of 0.70 (Hair, Sarstedt, Ringle, & Mena, 2012). This further confirmed a strong internal consistency of the constructs. Finally, the average variance extracted values of the three constructs were computed—(perceived) constraints (0.502), efficiency gain (0.702), and external pressure (0.719)—and all the values are greater than or equal to the minimum recommended value of 0.50 and thus have met the discriminant validity criteria (Koufteros, Babbar, & Kaighobadi, 2009).

**Table 4:** Factor analysis of the barriers and incentives for implementing FSMSs (*n* = 94)

Barriers to implement FSMSs	(Perceived) constraints (α = 0.852)	Efficiency gain (α = 0.890)	External pressure
			Industry competitiveness + regulatory and customer pressure (α = 0.959)
Lack of willingness by other supply chain partners to participate in the implementation of FSMS	0.798		
Lack of clarity about the benefits to be gained from implementing FSMS vis‐à‐vis required investment costs	0.783		
Lack of trained staff for technical and management aspects of FSMS	0.781		
Expensive and complicated task (i.e., there are economic, technological and legislation constraints)	0.729		
Resource‐intensive, require much administration and paper works which place a burden on companies	0.687		
Lack of complete, accurate, timely, and easily accessible information about the need for FSMS	0.646		
Not required by (non) governmental agencies	0.618		
Not familiar to customers and consumers	0.596		
Incentives to implement FSMSs			
FSMSs avoid duplication between processes		0.870	
FSMSs reduce product losses		0.862	
FSMSs increase operational efficiency		0.853	
FSMSs improve quality of management		0.803	
FSMSs streamline paperwork		0.797	
FSMSs attract new customers			0.932
FSMSs enhance market leadership			0.931
FSMSs improve market share			0.916
FSMSs improve relationships with customers			0.900
FSMSs provide customer assurances			0.879
FSMSs help access to new markets			0.876
FSMSs provide evidence of legal compliance			0.856
FSMSs enhance export competitiveness			0.830
FSMSs improve company image			0.777
FSMSs improve relationships with suppliers			0.700
FSMSs help avoid negative media attention			0.686
Kaiser–Meyer–Olkin measure of sampling adequacy	0.856		
Chi‐Square	2,173.875 (*p < .*0001)		

### 3.3 Econometric analysis

The decision to implement ISO 22000 is binary; thus, logistic regression was applied for the analysis. Several studies have applied a similar strategy to analyze the determinants of FSMSs adoption (e.g., Jin & Zhou, 2011). The main independent variables include the three latent variables identified above and variables measuring market orientation, product/process characteristics, and organizational readiness. Company size, education, ownership structure, and business years are controls.

The results in Table 5 have presented coefficients and odds ratio, in robust standard errors. The odds ratio shows the number of times a food processor is more or less likely to implement ISO 22000. The results, in general, support the hypothesized relationship in Figure 1. Market orientation is strongly and positively linked to ISO 22000 implementation. Also, the latent variable (perceived) external pressure is positively correlated with ISO 22000 implementation. On the other hand, constructs measuring (perceived) efficiency gain and barriers for implementing FSMSs are negatively correlated with the implementation of ISO 22000; however, only the former is significant. Organizational readiness is strongly correlated with ISO 22000 implementation. Likewise, product/process characteristics is an important predictor of ISO 22000 implementation; food processors engaged in meat, dairy, and/or fruits and vegetables processing are more likely to implement ISO 22000 than those enterprises primarily engaged in the processing of dry foods. Among the controls, company size and ownership structure are strongly linked with ISO 22000 implementation.

**Table 5:** Results of the logistic regression analyses on the determinants of ISO 22000 implementation

Variable	Coef. (*SE*)	Odds ratio (*SE*)
Market orientation (dummy, 1 = export; 0 = domestic)	1.699*** (0.634)	5.468*** (3.467)
(Perceived) constraints to implement FSMS (8 items, α = 0.852)	−0.702 (0.439)	0.496 (0.218)
(Perceived) efficiency gain (5 items, α = 0.890)	−1.523* (0.833)	0.218* (0.182)
External factors (11 items, α = 0.959)	1.620* (0.969)	5.053* (4.897)
Product/process characteristics (1 = Meat/dairy/F&V, 0 = other)	1.590** (0.645)	4.906** (3.178)
Organizational readiness (presence of Quality Assurance unit, 1 = yes, 0 = no)	2.709*** (1.101)	15.019*** (15.169)
Company size (# of employees, full‐time equivalent: 1, < = 50; 2, 51–250; 3, 250)	1.361*** (0.468)	3.900*** (1.824)
Education of General Manager (1 = postgraduate; 0, other)	1.682 (1.22)	5.378 (6.604)
Ownership structure (1 = family‐owned, 0 = other)	1.637** (0.813)	5.14** (4.178)
Years in business (dummy; 1,> 40)	0,240 (0.658)	1.272 (0.837)
Intercept	−7.1985*** (1.750)	0.001*** (0.001)
Pseudo *R*2	0.369	
Chi‐square	27.98	
Overall corrected prediction (%)	81.91	

Note

The independent variables were checked for multicollinearity; mean value of the VIF is reported at 3.03.

* *p* < .1; ^**^ *p* < .05; ^***^ *p* < .01.

### 3.4 Discussion

This study has sought to analyze the implementation of market‐based FSMSs in Lebanon, following the introduction of its first‐ever food safety law in 2016. The analysis was carried out by distinguishing market‐based FSMSs into public‐ and industry‐based. The findings show the public‐based international standards (i.e., ISO 22000, HACCP, and ISO 9001) being preferred by food processors in Lebanon. In fact, none of the processors had implemented any of the industry‐based FSMSs such as BRC, SQF, FSCC 22000, or IFS. This was surprising given the claims by past studies about the increasing role of industry‐based standards in the governance of global food supply chains (Henson & Humphrey, ) but may suggest the changing landscape of food safety governance across time, geography, and supply chains. Indeed, with the increasing harmonization of food safety regulations (through the work of FAO, WHO, and WTO), industry‐based FSMSs may be less preferred for firms participating in global food supply chains. Against this backdrop, the study offers some explanations about the dominance of public‐based international FSMSs in the context of Lebanon.

#### 3.4.1 The dominance of public‐based international FSMSs

Following the uncovering of several food safety scandals in recent times, there has been increased government oversight on food safety. For example, in 2014, the Lebanese Ministry of Health launched a series of inspection campaigns against food establishments and found 741 (27.5%) nonconforming samples (out of 2,716 samples collected from 1,077 food establishments). In this study, the findings show that none of the food processors in the survey had adopted any of the industry‐based (global) standards. Furthermore, of the food processors that had implemented public‐based international FSMSs, the majority of them were export‐oriented. The low adoption of industry‐based FSMSs may also be attributed to the domestic market environment, which is dominated by a large group of small (traditional) retailers (Bahn & Abebe, 2017). The small retail businesses may not exert enough pressure on food processors to implement industry‐based FSMSs that often go beyond the parameters of public‐based international FSMSs. However, in a competitive export market, participation is subjected to strict food safety requirements. The attractiveness of the public‐based international FSMSs by Lebanese processors can thus be explained by their increased level of participation in regional/international markets, such as Arab countries and Europe (IDAL, 2017), rather than a response to regulatory demands in the domestic market. A recent case study in Peru found no relationship between the performance of asparagus exports (both in volumes and value of exports) and the implementation of industry‐based FSMSs (Schuster & Maertens, 2015). This may be because industry‐based FSMSs are more stringent (Handschuch, Wollni, & Villalobos, 2013) and heterogeneous (ambiguous) and thus require higher compliance costs (Henson & Humphrey, 2010). For the Lebanese food companies, dominated by small to medium food processors, public‐based international FSMSs (e.g., ISO 22000) may be preferred to industry‐based FSMSs. The findings show, while 67% and 85% of food processors that implemented ISO 22000 were medium and large processors, respectively, small processors had increased the level of participation in the implementation of ISO 22000 (29%) compared to ISO 9001 (4%) and HACCP (19%). This may be true beyond Lebanon because, in a relatively short period, ISO 22000 has attracted over 161 national standards bodies (International Organization for Standardization, 2018).

#### 3.4.2 The effect of internal and external factors on the implementation of ISO 22000

Among the three public‐based international FSMSs, the study has paid attention to ISO 22000 due to its scale and scope of adoption in Lebanon. Most importantly, the study found relatively high participation of small processors in the implementation of ISO 22000. The econometric analysis revealed several factors attributed to the increased uptake of ISO 22000 in Lebanon.

First, the study found predominantly export‐oriented food processors have increased the odds of ISO 22000 implementation 5.5 times more than the domestically oriented food processors, implying that the implementation of public‐based FSMSs is largely driven by international trade. As discussed above, this is not surprising. Following the establishment of the “Euro‐Mediterranean partnership” and the creation of the free trade area between the EU and Southern Mediterranean countries, Lebanon's food export to the EU and Arab countries has improved significantly in recent times (Massoud et al., 2010).

Second, the latent variable “efficiency gain” was marginally significant and negatively correlated with ISO 22000 implementation; meaning, food processors have yet to see efficiency gains from FSMSs implementation. Also, the surveyed processors indicated a lack of willingness by other supply chain partners, lack of clarity about the potential benefits, high implementation and certification costs, and lack of knowledge and technical skills as barriers. Nonetheless, the application of ISO 22000 in Lebanon has increased. For example, there were only two ISO 22000 certifications awarded to a Lebanese company in 2007. But that figure grew to 143 by 2017 (International Organization for Standardization, 2018). In the Middle Eastern context, only United Arab Emirates and Saudi Arabia (196) have more ISO 22000 certifications than Lebanon. The findings suggest, with the increasing harmonization of food safety regulations and international trade, the cost of noncompliance would be too high to be ignored. Indeed ISO 22000 has become a de facto mandatory requirement for companies participating in regional/global markets (Hou, Grazia, & Malorgio, 2015).

Third, the benefits and constraints of implementing FSMSs can be attributed to several factors. Organizational readiness, an indicator to measure the degree of commitment, is positively and strongly correlated with ISO 22000 implementation. Prior studies have also documented the importance of organizational commitment for FSMS implementation (Qijun & Batt, 2016; Trienekens & Zuurbier, 2008). In the study context, food processors with a QA unit had 15 times higher chance of implementing ISO 22000 than organizations that did not have a QA unit. The education level of the manager, another proxy for organizational readiness (Kussaga, Jacxsens, Tiisekwa, & Luning, 2014), was also positively correlated with ISO 22000 implementation but statistically insignificant, perhaps due to a low variability of education level among the quality managers in the two subsamples. Likewise, product/process characteristics did have a strong and positive correlation with the implementation of ISO 22000. Food processors that are engaged in fresh produce such as meat, dairy or fruits/vegetables processing had 4.9 times higher chance of implementing ISO 22000 than those companies engaged in dry foods (e.g., cereals and baked goods). This was expected as fresh produce has higher chance of microbial or chemical contaminations than dry foods (Kirezieva et al., 2013). Among the control variables, only company size and ownership structure did have a significant correlation with ISO 22000 implementation.

Finally, among the external factors, industry competitiveness and customer requirements are important predictors of ISO 22000 implementation. This is consistent with Escanciano and Santos‐Vijande (2014) who documented similar findings among Spanish firms. A closer look at of the multi‐item scales retained to create this latent variable suggests the importance of economic incentives and industry competitiveness more than compliance costs and regulatory pressures.

## 4 CONCLUSIONS

Food safety continues to be a public health concern and a major development challenge. Although different types of FSMSs are proposed to address this issue, they continued to evolve and be scrutinized for their effectiveness and low uptake. Against this background, the study has analyzed the state of market‐based FSMSs and their determinants in the Middle Eastern context (Lebanon).

The study has identified public‐based international FSMSs as the dominant food safety governance regime in Lebanon. Second, based on a review of the literature (E. Chen et al., 2015; Escanciano & Santos‐Vijande, 2014; Herath & Henson, 2010; Qijun & Batt, 2016), the study presented a model that classified potential determinants of FSMSs implementation into internal and external factors to determine the drivers (or barriers) of implementing FSMSs, focusing on one of the widely recognized public market‐based schemes, ISO 22000. The study establishes market orientation as the most important driver for ISO 22000 implementation in Lebanon. Skills and financial resources are still important barriers to FSMSs implementation but appeared to be less relevant compared to the cost of noncompliance. Also, organizational readiness and product/processes characteristics are crucial to promote the implementation of ISO 22000. Regarding company size, small, medium, and large processors are involved in the implementation of the public‐based FSMSs, but at a varied level of intensity. The relatively increased level of participation by small processors in the implementation of ISO 22000 is encouraging and may suggest the attractiveness of ISO 22000 for small to large food processors.

In sum, the decision to implement the public‐based international FSMSs over the industry‐based FSMSs by Lebanese food processors appeared to be more of a strategic choice (driven by economic incentives) rather than statutory demands. The low uptake of industry‐based FSMSs in Lebanon may also be attributed to the characteristics of the domestic market, which is dominated by small retail businesses. Also, it is worth noting that resource intensiveness, increased administration and paperwork, lack of willingness by other supply chain partners, and the cost and complexity of implementing FSMSs continued to be barriers. Accordingly, support services and incentive schemes, particularly for small processors, would be vital to promote FSMSs in Lebanon. The Lebanese food retail sector is highly dispersed and controlled by a large group of small (traditional) retailers. Hence, public policy should focus on incentive schemes designed to attract large food retail chains to spearhead the implementation of industry‐based FSMSs and the development of integrated food supply chains. This is because, although public standards may be available to address food safety concerns, the adoption of industry‐based (private) standards is necessary to keep up with new production and distribution practices in which public‐based FSMSs may fail (or act slowly) to address current and future developments in food supply systems. Industry‐based FSMSs may be preferred to govern additional product and process attributes, including organic production, animal welfare, and environmental sustainability (Henson & Humphrey, 2010). Furthermore, the adoption of industry‐based FSMSs (led by large retail chains) is expected to drive the development of agrifood chains in developing countries by linking local producers to high‐value markets (Lee, Gereffi, & Beauvais, 2012).

## CONFLICT OF INTEREST

The authors declare that we do not have any conflict of interest.

## ETHICAL STATEMENT

The study conforms to the Declaration of Helsinki, USA, and/or European Medicines Agency Guidelines for human subjects. The study protocols and procedures were ethically reviewed and approved by the Institutional Review Board (IRB) at the American University of Beirut (AUB). This work does not involve any animal or human testing.
